# Squinting

**Published:** 1893-02

**Authors:** R. A. Chudleigh


### SQUINTING.

Each eye has six muscles, whereof two are used and placed just like the reins of a horse in harness. A frequent cause of convergent squint is paralysis of the external "reins," or rather, of the sixth nerve which regulates them. In this case there is nothing to oppose the two internal reins which lie on the sides of each eye nearest the nose, and consequently the eyes are drawn inwards. Let the child look at some object well on its right; the left eye will turn in towards the nose; but if the right eye cannot get out farther than the middle line, so that it looks straight forward while the left eye looks strongly to the right, then we may decide that there is a paralysis of the external rectus of the right eye. A similar test made by looking at an object well on the left will show if there be paralysis of the external muscle of the left eye. If no paralysis be thus revealed, the squint is probably due to over-contraction of the internal muscles. The other cause is, however, the most likely one, for it is not uncommon, is very noticeable, and may well be a sequel of whooping cough. Another possible cause is long sight. The effort to focus on a near object is always naturally coupled with convergence of the eyes on that object. In long-sight an over-effort is required, and an over-convergence of the eyes, which amounts to an inward squint, is the consequence. But this squint is not very noticeable, nor would it be connected with the whooping cough, though it specially affects young children. The foregoing is the best information I can give on this subject, and I regret that I cannot go on to advise some treatment. Except in those cases that get well of their own accord (and as six years have elapsed this does not seem to be one of them) the treatment requires the aid of an ophthalmic surgeon. In some cases prismatic lenses are prescribed ; in other cases the long-sight must be corrected by suitable spectacles ; in others, a tendon is divided ; in others, the muscles are "advanced" or re-adjusted. If the patient live near Birmingham take her to the eye-hospital, if in London get a recommendation to Moorfields.—R. A. CHUDLEIGH, M. D.

